# Anatomy (Double Number)

**Published:** 1893-03

**Authors:** 


Anatomy (Double Number). By Fred J. Brockway, M.D., Assistant Demonstrator of Anatomy, College of Physicians and Surgeons, New York, and A. O'Malley, M.D., Instructor in Surgery, New York Polyclinic. Being Volume I. of the Students' Quiz Series, edited by Bern B. Gallaudet, M.D., Demonstrator of Anatomy, College of Physicians and Surgeons, New York; Visiting Surgeon, Bellevue Hospital, New York. Pocket-size 12mo, 367 pages, 15 illustrations. Limp cloth, $1.75. Published by Lea Brothers & Co., Philadelphia.

In this presentation of modern anatomy, the authors and editor have done their work well. The essentials of the subject—the knowledge that will be most useful to the student and general practitioner—have been selected and arranged in a concise and compact form. A Glossary has also been prepared, in the hope of promoting a correct pronunciation of anatomical terms.

The typography, however, could be improved upon. In printing text-books a type that would be less trying upon the eyes should be employed. W.

